# *Yersinia pseudotuberculosis* Infection Complicated with Bacteremia in a 10-Month-Old Boy

**DOI:** 10.1155/2020/8846511

**Published:** 2020-11-27

**Authors:** Takuro Kamura, Yuhei Tanaka, Naoya Tsumura, Takashi Ohya, Yuki Okamatsu

**Affiliations:** ^1^Department of Pediatrics, Aso Iizuka Hospital, 3-83 Yoshio Town, Iizuka 820-8505, Japan; ^2^Department of Pediatrics and Child Health, Kurume University School of Medicine, 67 Asahi Town, Kurume 830-0011, Japan

## Abstract

*Yersinia pseudotuberculosis* (*Y. pseudotuberculosis*) infection complicated with bacteremia rarely occurs. *Y. pseudotuberculosis* infection is also known to produce various symptoms similar to Kawasaki disease (KD) due to the production of *Y. pseudotuberculosis*-derived mitogen (YPM), an exotoxin with superantigen activity. Moreover, it causes terminal ileitis and is responsible for appendix swelling. Here, we report a case of *Y. pseudotuberculosis* infection in a 10-month-old boy who was brought to our hospital due to fever, watery stool, and poor vitality. Abdominal echocardiography revealed wall thickening of the entire colon and appendix swelling; therefore, he was admitted and treated with antibiotics for bacterial enteritis or appendicitis. After the antibiotic administration, facial skin rashes and hand and foot edema developed. However, he had 5/6 major symptoms of KD and was diagnosed with *Y. pseudotuberculosis* infection because of its presence in the blood and stool cultures. Thereafter, antibacterial therapy improved his symptoms and increased the inflammatory response. After his hospital discharge, the skin on his fingers showed desquamation like that of KD. *Y. pseudotuberculosis* infection should be considered as a differential disease in KD, terminal ileitis, and appendicitis. Furthermore, its infection route and culture methods should also be carefully considered.

## 1. Introduction

*Yersinia* species are anaerobic Gram-negative bacilli belonging to the Enterobacteriaceae family, which develop bacterial enteritis by ingesting contaminated mountain or well water. Among them, *Yersinia pseudotuberculosis* (*Y. pseudotuberculosis*) is known to cause various symptoms in children, including Kawasaki disease (KD). Herein, we report a case of an infant diagnosed with enteritis and bacteremia due to *Y. pseudotuberculosis* based on blood and stool culture results.

## 2. Case Presentation

Written informed consent was obtained from the guardian of the patient for publication of this case report and accompanying images.

**Figure 1:**
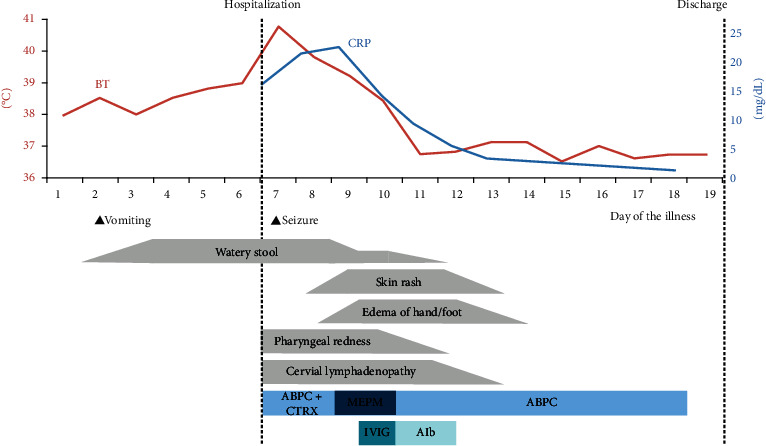
Clinical course of the patient. BT, body temperature; CRP, C-reactive protein; ABPC, ampicillin; CTRX, ceftriaxone; MEPM, meropenem: IVIG, intravenous immunoglobulin; Alb, albumin.

**Figure 2:**
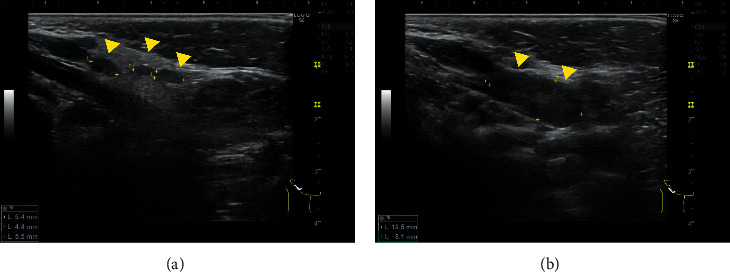
Cervical ultrasonography revealing mild reactive lymphadenopathy.

**Figure 3:**
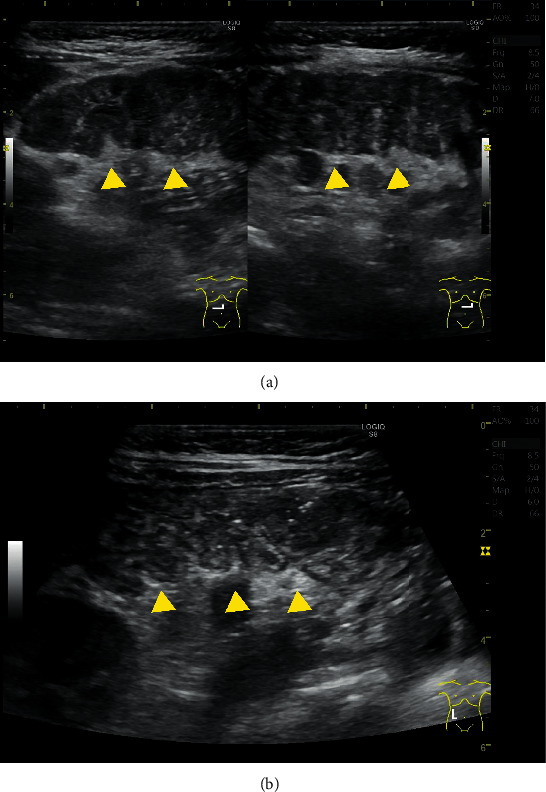
Abdominal ultrasonography revealing intraperitoneal lymphadenopathy, wall thickness of the entire colon.

**Figure 4:**
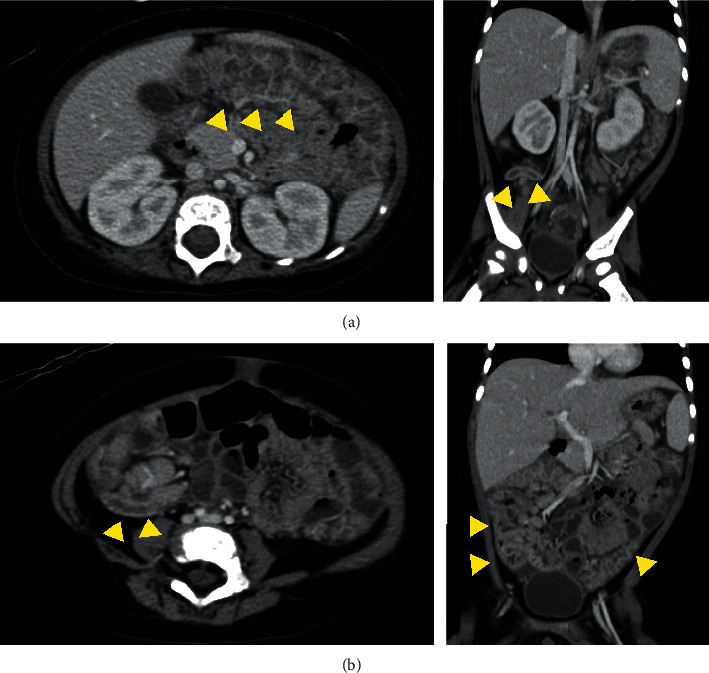
Abdominal computed tomography revealing pitting-like wall thickness of the entire colon, mesenteric lymphadenopathy of the right lower abdomen, and enlarged appendix.

A 10-month-old boy was brought to our hospital with fever, watery stool, and poor vitality. He developed fever from the 1^st^ day, water stool (15–20 times a day) from the 2^nd^ day, and poor vitality from the 7^th^ day of illness. Vomiting occurred twice only on the 2^nd^ day. He was hospitalized on the 7^th^ day due to persistent symptoms and poor oral intake and vitality. He was previously healthy, with no recent history of travel, contact with sick individuals, or exposure to pets. His body temperature was 40.8°C; pulse, 184 bpm (normal); blood pressure, 121/66 mmHg; and respiratory rate, 36 breathes/min with an oxygen saturation of 99% on room air. On physical examination, he was nonresponsive and had cervical lymphadenopathy, increased bowel sounds, and peripheral cold sensation. No other lymph node swelling, rash, or edema was observed (Figure 1). Initial laboratory results revealed elevated inflammatory markers (white blood cell (WBC) count, 15,750/*μ*L; C-reactive protein (CRP), 16.83 mg/dL; and procalcitonin, 8.4 ng/mL) and hyponatremia (130 mmol/L). Cervical ultrasonography revealed mild reactive lymphadenopathy (Figure 2). Abdominal ultrasonography (Figure 3) and computed tomography (CT) (Figure 4) revealed pitting-like wall thickness of the entire colon and an enlarged appendix.

Initially, estimating the pathogenic bacteria and infection route was difficult through interviews; therefore, a broad-spectrum antibiotic therapy was administered (ampicillin: ABPC, 200 mg/kg/day + ceftriaxone (CTRX), 100 mg/kg/day). On the same day, 2-3-min convulsions occurred with upturn of the eyeball and poor complexion. Convulsions were identified as systemic tonic, without left-right differences. Cranial CT revealed no abnormal findings such as cerebral edema or neoplastic lesions, and cerebrospinal fluid examination revealed normal leukocyte levels or hypoglycemia (cerebrospinal fluid (CSF): glucose level of 73 mg/dL as compared to simultaneous blood glucose level of 113 mg/dL, protein of 13 mg/dL, and cell count of 4 cells/*μ*L with 58% cells being neutrophil).

On day 9 of illness, symmetrical pale erythema without blisters and bulges were observed on the patient's face and trunk. Follow-up laboratory results on the same day were as follows: WBC, 13290/*μ*L and CRP, 23.3 mg/dL. The blood culture on the 7^th^ day of the illness (hospitalization date) was reported to be positive for Gram-negative rod and was identified as *Y. pseudotuberculosis.* On the next day, the guardian was reinterviewed, revealing that they had been using spring water from nearby valleys for domestic purposes. The spring water was boiled and then used to melt the formula (artificial milk). Antibiotics were changed to meropenem (MEPM, 120 mg/kg/day), and intravenous immunoglobulin (IVIG, 500 mg/kg/day) was administered for hypogammaglobulinemia (443 mg/dL). On day 10 of illness, the fever subsided. On day 11, antibiotics were changed to ABPC (200 mg/kg/day) based on the antibiotic sensitivity to *Y. pseudotuberculosis*. Intravenous albumin (10 g/dose/day) was also administered because of body weight gain and edema associated with hypoalbuminemia. These treatments gradually improved his watery stool, rashes, and cervical lymphadenopathy. Oral intake became possible on day 12 of illness, antibiotics were continuously infused until day 18, and he was discharged on day 19. During the hospitalization, no symptoms of acute renal failure were suspected. Finally, *Y. pseudotuberculosis* was detected from the stool culture and loop-mediated isothermal amplification (LAMP), and *Y. pseudotuberculosis*-derived mitogen (YPM) antibody was also increased, resulting in the diagnosis of enteritis and bacteremia due to *Y. pseudotuberculosis*. After discharge, he presented desquamation of both hands on the 30^th^ day of illness. He has been under follow-up care without coronary artery lesions.

## 3. Discussion

Yersiniosis is a zoonotic infection occurring in domestic and wild animals; humans are considered incidental host that do not contribute to the natural disease cycle. The genus *Yersinia* includes 18 species, three of which are important human pathogens: *Yersinia pestis*, *Yersinia enterocolitica*, and *Y. pseudotuberculosis*. *Yersinia* infections are widespread globally, but most frequently reported in Europe [1]. It was first reported in Japan in 1972, and since then, sporadic cases and outbreaks of childhood diarrhea have been reported. *Yersinia enterocolitica* infections are common in eastern, whereas *Y. pseudotuberculosis* infections are common in the western region in Japan, and are closely related to the carriage of wild animals in each region [2]. Transmission of yersiniosis is largely food-borne and occasionally water-borne (untreated landslides and well water). *Yersinia* is isolated from animals such as pigs, dogs, cats, rabbits, and monkeys, as well as from rodents such as rats and raccoons that contaminate the water surface. In addition, as it is widely distributed in wildlife, meat and raw milk contaminated with feces also cause infections. Its incubation period is 2–20 (average, 8) days, and infants may become sick even with small doses.

Acute yersiniosis presenting with right lower abdominal pain, fever, vomiting, leukocytosis, and mild diarrhea [3] may be misinterpreted as acute appendicitis. In typical cases, abdominal ultrasonography and CT reveal thickening of the terminal ileum and enlarged lymph nodes around the intestinal tract [4]. In this case, similar imaging finding and appendix swelling were also revealed; thus, inflammatory bowel disease and acute appendicitis were suspected as differential diagnosis. The prevalence of acute appendicitis in young children is low, i.e., 1–6/10000 in children from birth to 4 years, but 19–28/10000 in those aged 4–14 years [5]. Yersiniosis should be considered as a differential disease when diagnosing terminal ileitis and appendicitis in young children, especially up to 4 years old.

Furthermore, *Y. pseudotuberculosis* infection has been known to manifest various symptoms including those of KD, i.e., 13% of patients have been reported to have met the diagnostic criteria of KD [6]. In addition, 10% of patients with KD have also been reported to test positive for anti-YPT or anti-YPM antibodies, and the positive group had a higher incidence of coronary sequelae (18% in the positive group vs. 1% in the negative group) [7]. Moreover, among patients with *Y. pseudotuberculosis* infection, frequencies of diarrhea and abdominal pain in the family and use of natural water sources were reported to be significantly higher in the *Y. pseudotuberculosis* antibody-positive group [8]. In KD, especially those with gastrointestinal symptoms, *Y. pseudotuberculosis* infection should be suspected, and therefore, culture and antibody titration should be performed. Moreover, even in patients diagnosed with *Y. pseudotuberculosis* infection, IVIG should be administered when antibacterial treatment is ineffective and meets the diagnostic criteria for KD, and coronary artery evaluation with echocardiography should also be performed regularly.

*Yersinia* is generally difficult to detect in culture. For efficient detection, a low temperature (25°C–29°C) should be used with a special selective medium, for example, efsulodin-irgasan-novobiocin (CIN) agar or CHROMagar *Yersinia enterocolitica* (CAYe) [9]. Therefore, when submitting stool culture, “*Yersinia*” should be clearly indicated as the target bacterium. Nevertheless, some case reports demonstrated difficulty in identifying *Yersinia*, resulting in not achieving a definitive diagnosis. In this case, the diagnosis of yersiniosis was possible from culture results; however, it should be noted that even if the culture is negative, serum antibody titer measurement or LAMP-based assay can be used to diagnose yersiniosis [10].

This case was complicated with bacteremia due to *Y. pseudotuberculosis*. Although similar cases have been reported, most of them are adult patients with underlying disorders, such as diabetes mellitus, liver cirrhosis, iron overload, malignant tumor, and after organ transplantation [11–14]. Very few cases of bacteremia due to *Y. pseudotuberculosis* have been reported in children without risk factors [14]. *Y. pseudotuberculosis* migrates to the mesenteric lymph node across Peyer's patch in the small intestine after ingestion [15]. Considering the findings of total colon thickness and mesenteric lymphadenopathy, *Y. pseudotuberculosis* possibly migrated further systemically from the mesenteric lymph nodes to the blood system.

Furthermore, symptoms did not improve despite of the initial antibiotic therapy. Convulsions even occurred for several minutes on the 7^th^ day of illness. Based on the CSF examination results, encephalitis was considered to be unlikely the cause of convulsions. Although acute encephalopathy has been reported to occur as a rare complication of *Y. pseudotuberculosis* infection [16, 17], mild encephalopathy cannot be denied because head MRI examination was not performed. However, postconvulsive consciousness was clear, without recurrent convulsions and prolongation of consciousness disorder; thereby, the patient is suspected to have simple febrile seizure only. Antibiotic therapy is effective for sepsis, but its effectiveness for other clinical symptoms is unknown. Antibiotics therapy was considered ineffective against YPM, which has a superantigen activity, and causes various types of systemic damage [18]. Subsequently, early antibiotic therapy finally succeeded, although the mortality rate of bacteremia due to *Y. pseudotuberculosis* was reported to be up to 75% [12].

In addition, his guardian stated in an interview during the hospitalization that they do not take natural water, but further interview revealed that “spring water from a nearby valley” had been used to melt the patient's formula (artificial milk).

*Y. pseudotuberculosis* infection rarely occurs and should be considered as an important differential disease in KD or appendicitis. The route of infection and culture methods should also be carefully considered, and serum antibody titers should be measured if *Y. pseudotuberculosis* infection is suspected.
